# Burnout and moral distress among school nurses during the COVID-19 pandemic: a brief report

**DOI:** 10.3389/fpubh.2025.1750354

**Published:** 2026-01-09

**Authors:** Nakia C. Best, Cassidie S. Thomas, Bosny Pierre-Louis, Annabelle Wu, Amanda Chang, William Bevens, Shannon Baker Powell

**Affiliations:** 1 Sue & Bill Gross School of Nursing, University of California, Irvine, Irvine, CA, United States; 2 Novion Analytics, Durham, NC, United States; 3 Joe C. Wen School of Population & Public Health, University of California, Irvine, Irvine, CA, United States; 4 Department of Psychiatry, University of California, San Diego, La Jolla, CA, United States; 5 College of Nursing, East Carolina University, Greenville, NC, United States

**Keywords:** burnout, COVID-19, moral dilemmas, moral distress, resilience, school nurse

## Abstract

**Introduction:**

School nurses play a critical role in supporting student health but faced unprecedented burdens during the COVID-19 pandemic, including increased workloads, rapidly changing guidelines, and heightened risk of exposure. These pressures contributed to burnout and moral distress (MD) threatening workforce stability. This study aimed to quantify burnout among school nurses during the pandemic and examine its relationship with moral distress.

**Methods:**

A cross-sectional, mixed-methods survey was administered in December 2022 to school nurses in a large, diverse region of California. The survey utilized the Mini-Z to measure burnout, Moral Distress Thermometer to measure moral distress, and school nurse moral dilemmas. The survey also included open-ended questions about school nurses' biggest challenges, supports for their practice, and sources of hope. Quantitative data were analyzed using *t*-tests and correlations, and qualitative data were analyzed through content analysis.

**Results:**

Forty percent of school nurses reported burnout. Burned-out nurses had significantly higher moral distress scores (*p* < 0.01), and burnout severity correlated with higher moral distress (*r*_s_ = 0.26, *p* = 0.045). Burnout was most strongly associated with systemic barriers, such as the inability to provide preventive care. Qualitative findings further highlighted overwhelming workloads and systemic challenges as primary stressors, while peer support was identified as an important source of hope.

**Conclusion:**

The pandemic was associated with significant burnout and moral distress among school nurses, largely driven by systemic constraints. Sustaining this essential workforce and protecting student health will require systemic solutions including increased staffing, improved resource allocation, and robust organizational support to enhance nurse wellbeing and resilience.

## 1 Introduction

School nurses are healthcare providers in the school setting who play a critical role in promoting child and adolescent health, wellbeing, and academic success (1, 2). Rooted in public health, school nurse practice includes support for student physical, emotional, behavioral, and social health to maintain or improve student outcomes (3). They accomplish this through preventive health programs, direct acute care, chronic health management, care coordination, and education programs for students, families, and school staff (1).

To ensure school nurses are available to provide these health care services and more, students should have access to a registered school nurse every school day, all day (1, 2). Unfortunately, only 65.7% of schools in the U.S. have access to a full-time school nurse (4). Also, school nurses may be responsible for students from multiple grade levels across numerous school sites with caseloads ranging from hundreds to thousands of students per nurse (i.e., school nurse-to-student ratio) (5). Within the school nursing caseload, various factors also impact the overall intensity of their work, including the complexity of student health needs and the support and resources available to the school nurse (6). Due to these factors that increase the workload burden, school nurses must prioritize immediate health crises for individual students, leaving insufficient time for population-wide preventive health initiatives (7).

Healthcare professionals experienced unprecedented psychological burdens during the COVID-19 pandemic, with significant implications for healthcare system stability and quality of care. School nurses, a critical yet understudied healthcare workforce, play essential roles in mitigating social vulnerability by addressing student health needs that influence families and broader school communities. During the COVID-19 pandemic, students' lives were significantly disrupted by school closures when they lost access to services for acute and chronic health conditions, behavioral health services, and reliable nutrition (8, 9). School nurses, like other pediatric healthcare professionals, pivoted the way they delivered services to address students' needs. However, the rapidly evolving guidelines and policies in response to the pandemic, left school nurses feeling overwhelmed and as if they were falling short (10). The constant changes, feelings of not doing enough, job-related threats and harassment, additional COVID-19 related duties, and moral conflicts between safety protocols and student care needs, resulted in unintended mental health consequences, notably burnout (11–13). Despite their critical role in student healthcare delivery, school nurses' mental health outcomes during the pandemic remain poorly understood, representing a significant gap in healthcare professional wellbeing research. Inadequate support for school nurses during the COVID-19 pandemic is therefore not only a school nurse or school health concern, but also a broader public health issue, as school nurse burnout may compromise the availability of school-based health services, exacerbate social vulnerability among students, and weaken the capacity of schools to respond to public health emergencies.

Burnout unfolds as a progressive process where healthcare workers experience declining morale and reduced enthusiasm for their roles, typically precipitated by overwhelming job demands, obstacles that prevent meaningful engagement with their professional mission, or fundamental disconnects between their core values and their organization's practices (14). Burnout is especially common in nursing, where professionals face the dual burden of navigating emotionally demanding patient interactions while operating within systems characterized by heavy caseloads and time constraints, which often conflict with their core mission of delivering compassionate, comprehensive healthcare (15). This creates a cascade of negative effects that extends beyond individual school nurses, encompassing student welfare and the entire school community, while simultaneously diminishing job performance and driving school nurses away from the profession (16).

Moral distress (MD) reflects the experience of being blocked, by contextual constraints or one's own limitations, from acting on what one's professional judgment and personal values deem right (17–19). For school nurses, moral distress emerges when policies, resource shortages, or personal constraints prevent action consistent with their clinical judgment and core values (10, 17, 20). Moral dilemmas, such as being unable to provide care due to a lack of time or being concerned about students with chronic health conditions not receiving needed care, may manifest for school nurses due to their role and particular work environment (21). This was found true in recent qualitative studies exploring school nurse perspectives about the impact of the pandemic on school nurses and school nurse health services in California, U.S. (10) and Colorado, U.S. (22).

This study aimed to assess burnout and its relationship with moral distress among school nurses during the COVID-19 pandemic. Specifically, we sought to: (1) quantify burnout levels among school nurses who worked during 2020–2022, (2) examine correlations between burnout and moral distress, and (3) identify key challenges and coping mechanisms through qualitative analysis.

## 2 Methods

### 2.1 Study design, setting, participants

We administered a cross-sectional survey in December 2022 to school nurses who worked in K-12 schools in a large, diverse urban-suburban region of California between 2020 and 2022. The region is home to a large and ethnically diverse student population, with over 470,000 students during the study period.

### 2.2 Measurement

The survey included school nurse demographic information and questions about virtual school health services offered during the pandemic, COVID-19 related activities, and nurse-specific duties provided (23). To assess school nurse burnout, we used the Mini Z's single item measure of burnout (24). This item asks the participant about their definition of burnout on a scale of 1–5. A score of 3–5 signals burnout. The Moral Distress Thermometer (MDT) (19), a one-item, 11-point scale (0-none to 10-worst possible), was used to measure moral distress in the past month. We also included 14 questions (5-point Likert scale-strongly agree to strongly disagree) developed by Powell et al. (21) to measure moral dilemmas that school nurses may encounter. We included three open-ended qualitative questions about school nurses' biggest challenges (23), what would help them do their job, and what were their sources of hope and encouragement during the pandemic (25). The number of participants who responded to questions varied; the sample size (*n*) is reported in the results for each question. This study was deemed exempt by the Institutional Review Board of the University of California, Irvine (# 1420).

### 2.3 Data analysis

The study employed a mixed-methods approach. Descriptive statistics were computed for demographics and mental health measures (burnout, moral distress, moral dilemmas). We created a dichotomous variable for the outcome of burnout (scores 3–5 = yes, scores 1–2 = no). Independent sample *t*-tests were used to compare moral distress and moral dilemmas between nurses in these two categories of burnout. The “not burned out” group was not normally distributed; thus, the non-parametric Mann–Whitney *U* test was applied. For those who were burned out, we used Spearman correlation to examine associations with moral distress and Pearson correlation to examine correlations with each moral dilemma. All tests of hypotheses were two-tailed, and a *p*-value < 0.05 was considered statistically significant. No adjustment was made for multiplicity. All analyses were performed using SAS^®^ software (Version 9.4 or higher).

Alongside these quantitative analyses, we conducted an inductive content analysis of the qualitative data (26–28) to capture patterns and emerging themes not reflected in the numerical measures above. Three team members independently analyzed the data (NCB, CST, and AW), then discussed until consensus was reached and confirmed to have been reliably interpreted.

## 3 Results

### 3.1 Demographic characteristics

A total of 154 school nurses participated in the survey, representing approximately 95% of school nurses employed in the region at the time. The school nurses were predominantly female (97%), bachelors educated (48%) and worked full-time (90%) in the public school (87%) setting. On average, they had 16 years of nursing experience and 8 years of school nurse experience (Table 1).

**Table 1:** School nurse characteristics (*n* = 154).

**Characteristic**	***n***	**%**
**Gender**		
Female	149	97
Male	5	3
**Age**		
≤ 30	33	21
31–40	38	25
41–50	32	21
51–60	33	21
≥61	18	12
**Highest degree earned**		
Diploma	17	11
Associates	3	2
Bachelors	74	48
Masters	55	36
Doctorate	1	1
No response	4	3
**California school nurse credential**		
Yes	98	64
No	55	36
No response	1	1
**Additional certifications or licenses**		
Public health nursing	49	32
Nationally certified school nurse	2	1
Nurse practitioner	3	2
Other	14	9
Multiple	17	11
No response	69	45
**School setting**		
Public	134	87
Charter	3	2
Private	11	7
Multiple	5	3
No response	1	1
**Grade level and populations served**		
Head start, Pre-K, TK, nursery	69	45
K-5th/6th grade	117	76
6th-8th grade	89	58
9th-12th grade	56	36
Children with disabilities	57	37
Adult transition	7	5
**Nursing experience**		
< 10 years	44	29
10–15 years	26	17
>15 years	78	51
No response	6	4
**School nurse experience**		
< 10 years	59	38
10–15 years	30	19
>15 years	62	40
No response	3	2
**Number of students**		
< 500	57	37
500–1,000	45	29
1,001–2,000	16	10
2,001–3,000	3	2
3,001–4,000	0	0
4,001–5,000	0	0
>5,000	1	1
No response	32	21
**Number of schools**		
1 school	107	69
2 schools	17	11
3 or more schools	17	11
No response	13	8
**Employment**		
Full-time	139	90
Part-time	12	8
Other	3	2
**Lead/coordinating nurse**		
Yes	36	23
No	118	77

### 3.2 School nurse health services

School nurses (*n* = 154) reported school health services they provided (Table 2), including pandemic preparedness (95%), chronic health management (89%), virtual services (70%), and other important services (54%). Specifically, most school nurses reported answering calls from parents/community (95%), educating/training school staff (93%), working on student care plans (89%), assisting students in managing chronic health conditions (87%), and conducting contact tracing (80%).

**Table 2:** School health services provided by school nurses during the pandemic (*n* = 154).

**Category**	**School nurse health services**
Pandemic preparedness	Answer parent/community phone calls
	Educate/train staff
	Outreach to students at risk
	Classes/videos about COVID-19
	COVID-19 screening of students, school staff and visitors
	Update/develop return to school plans
	Disseminate health department updates
	Meetings with administrators
	Review school health data
	Assist with deliveries to students
	COVID-19 testing
	Contact tracing
	Vaccine clinics
Chronic health management	Assist students
	Medication/equipment return to families
	Work on student health care plans and other SN duties
Virtual services	Office hours
	Support groups (e.g., stress, chronic condition management)
	Meetings (e.g., IEPs, physicians, screening referral follow-up)
Other	Teach or provide staff self-care
	Classes/videos about dealing with stress
	Update/develop other school health policies
	Develop/maintain professional school nurse webpage
	Participate in professional development

SN, school nurse; IEP, individualized education program.

### 3.3 Burnout and moral distress

**Figure 1:**
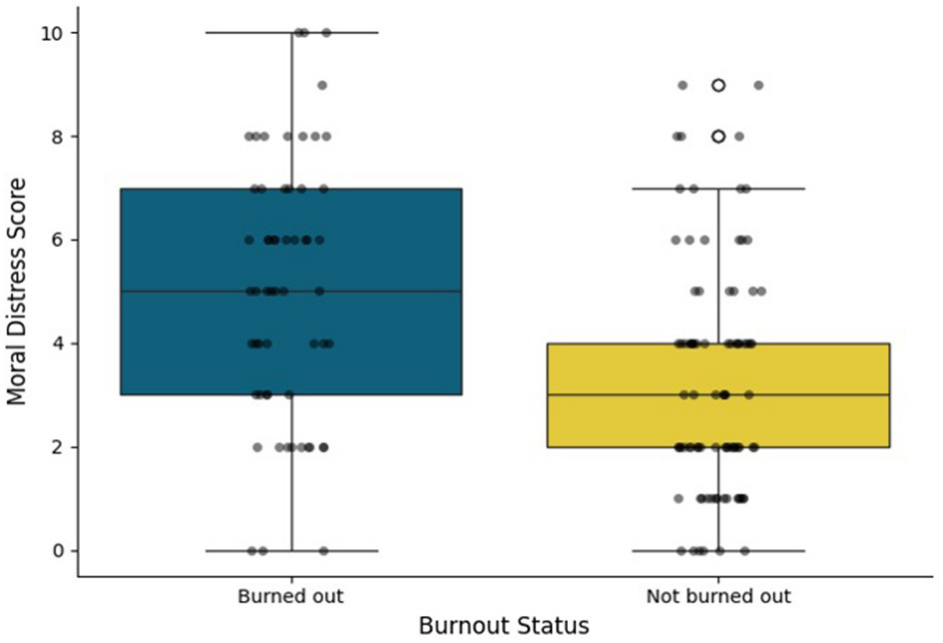
Boxplots of moral distress scores by burnout status among school nurses (*n* = 145).

Of the 149 school nurses who responded to the burnout question, 60 (40%) reported experiencing burnout. The overall mean moral distress score (*n* = 145) was 4.01 ± 2.51 (i.e., uncomfortable) on a 0–10 scale. School nurses who experienced burnout showed significantly higher moral distress scores compared to colleagues who did not experience burnout (mean = 5.05 ± 2.55, uncomfortable/distressing vs. mean = 3.29 ± 2.25, mild/uncomfortable; *p* < 0.01; Figure 1). Among burned out nurses, higher burnout severity was significantly associated with higher moral distress levels (*r*_s_ = 0.26, *p* = 0.045).

### 3.4 Burnout and moral dilemmas

First, we compared moral dilemma scores between burned out school nurses and those nurses who were not burned out. For each moral dilemma, nurses who were burned out were more likely to perceive and agree with the statements describing moral conflicts and constraints in their professional roles. For all variables except “unable to achieve goals for student due to family situation,” the difference between groups was significant (*p* < 0.05). Among burned out school nurses, burnout severity showed negative correlations with most moral dilemma items (Table 3). The strongest moral dilemma correlations with burnout were found for “unable to provide preventive care” (*r* = −0.35), “unable to provide care due to lack of school resources,” and “unable to provide care due to lack of referral services” (both *r* = −0.29).

**Table 3:** Correlations between burnout and school nurse moral dilemmas.

**Moral dilemmas**	**Correlation with burnout**
Unable to provide preventive care	−0.35
Unable to provide care due to lack of school resources	−0.29
Unable to provide care due to lack of referral services	−0.29
Unable to provide care due to workload	−0.24
Concern students with chronic illness do not receive needed care	−0.21
Unable to provide care due to lack of time	−0.19
Pressure from administration	−0.18
Unable to address staff requests due to lack of time	−0.18
Not enough time to provide care to students with chronic illness	−0.16
Unable to provide case management due to workload	−0.15
Unable to address family requests due to lack of time	−0.11
Pressured to not interrupt class to provide needed care	−0.11
Unable to achieve goals for student due to family situation	−0.08
Don't have a private space	0.15

### 3.5 Biggest challenges school nurses reported

School nurses (*n* = 132) provided qualitative responses detailing their biggest professional challenges, represented by five categories: school nurse workload (48%), student and family issues (34%), COVID-19 related challenges (31%), systems issues (11%), and nurse mental health (8%). For example, school nurses expressed challenges about their workload related to staff shortages, high nurse-to-student ratios, high workload burden, and expansion of their role due to the pandemic, “2 of my campuses are exclusively special education… this population just keeps growing. My caseload appears small, but my workload is huge with IEP assessments, reports and meetings, staff trainings, coordinating with families and service providers, keeping up on medical advancements and changes in legal issues.” Another nurse reported they had “more students with significant emotional needs some as a result of being home alone, unsupervised during COVID trying to keep up their studies which was stressful to our students. Students also took on/witnessed the stress in their homes as a result of financial pressures, lack of jobs... and students did not get the parental support as before the pandemic.” Additionally, another nurse stated, “On a personal level... burnout... recovering from COVID burden. Dealing with the pandemic as a school nurse was traumatic because we functioned as a clinic and the burden fell on us to triage, contact trace and be the medical experts. It felt like I was going into a battlefield sometimes.”

### 3.6 Identified supports for school nursing practice

Overall, school nurses (*n* = 130) reported supports and resources that would be beneficial to their practice related to resources and professional support (36%), decreased workload (31%), improved relationships with leadership, recognition, and supportive environment (19%), increased hours and compensation (18%), and improved student/family support and communication (9%). Most school nurses described the need for additional staff (e.g., health aides/clerks in classrooms, administrative assistants in the health office, other licensed nurses) to ensure school campuses had healthcare coverage during the entire school day: “... as the only one [nurse], if I go down, it all goes down!” They also requested more hands-on training for themselves and health aides “to improve their confidence and abilities to provide direct care to students.” Workload also emerged as a recurring issue for nurses: “I need more help with the number of diabetic [students] that I have. I am managing six at this time.” “[A] lower caseload would really assist me to fully be engaged and create thoughtful care plans for my students.”

### 3.7 Sources of hope for school nursing practice

School nurses (*n* = 118) contributed qualitative responses articulating the sources that gave them hope during the pandemic: support from others at work (53%), support from others outside of work (25%), influence on mindset (19%), faith, spirituality, and church (15%), and appreciation from students, families, and community (12%). Overwhelmingly, nurses were grateful for their colleagues, including other nurses, administrators, direct supervisors, and office staff: “My fellow school nurses, my admin staff and front office support staff. Very scary at the beginning with the amount of deaths in the community that the district serves.” Nurses were also supported by others outside of their workplace, including family and friends. Additionally, nurses found ways to shift their mindset to be positive and productive and find their meaning or purpose in their work: “school nurses went on battle mode. I found my strength in knowing I was helping staff and students.”

## 4 Discussion

We conducted this study in December 2022, during the later phase of the COVID-19 pandemic and several months before the official end of the U.S. federal public health emergency in May 2023 (29). Our study revealed significant burnout and moral distress among school nurses during the COVID-19 pandemic.

School nurses are essential to the health and wellbeing of students and the broader school community (1, 2). Despite the challenges school nurses encountered during the pandemic, they provided a wide range of critical services, including pandemic preparedness, chronic health management, and health education, that directly supported student and community health and safety. Lessons learned from the COVID-19 pandemic can inform and strengthen the school nurse role, making future emergency responses more effective and streamlined to proactively establish administrative and community support. We recommend school administrators proactively integrate school nurses into emergency planning and policy development (30), leveraging school nurse expertise to guarantee systems are in place before a crisis occurs. Including school nurses in policy and planning ensures there is someone “at the table” who is knowledgeable in and has access to current medical and public health information (10, 16). This not only keeps the students and community safe but also empowers school nurses to be actively involved and collaborate with school administrators.

Our finding of a 40% burnout rate among school nurse participants was similar to other studies. In national surveys conducted between 2021 and 2024, which surveyed school nurses, nurses overall, and healthcare professionals overall, 45%−50% of participants reported feelings of burnout (12, 31, 32). This pattern points to serious threats to workforce sustainability and underscores the urgent need for structural changes that support school nursing practice and protect the mental health of healthcare professionals. With only 65.7% of schools in the U.S. having access to a full-time school nurse (4), the loss of experienced practitioners due to burnout could have cascading effects on student health and academic outcomes. The powerful description of pandemic nursing as feeling “like a battlefield” underscores the traumatic nature of their experience and suggests that recovery from this period may require targeted interventions addressing both individual and systemic factors.

The relationship between burnout and moral distress appears cyclical: as nurses experience moral distress from being unable to provide optimal care, their risk of burnout increases, which further reduces their capacity to address student needs, perpetuating the cycle. This finding aligns with broader healthcare literature (33, 34), but takes on particular significance in the school setting, where nurses often work in isolation without the peer support systems available in traditional healthcare environments (35, 36). Strategies such as attending regular interdisciplinary meetings and shared professional development (e.g., principals, teachers, counselors, etc.) and building relationships with school staff and community partners can counteract working in isolation. Team meetings and joint workshops with other school and healthcare professionals helps to build mutual understanding and respect, and fosters information sharing and collaboration (37, 38).

Our findings revealed that the strongest moral dilemma correlations with burnout were related to resource constraints, specifically, the inability to provide preventive care, lack of school resources, and insufficient referral services. These results suggest that moral distress is primarily driven not by individual inadequacies but by systemic barriers or limitations to provide adequate support for school nursing practice. These findings align with previous studies that overwhelmingly found that moral distress is rooted in systemic barriers such as shortages in resources, policy constraints, and lack of support (34, 39).

During the pandemic, a concerning pattern emerged where any “health-related” responsibility defaulted to school nurses, regardless of whether it required clinical expertise. This scope creep, while understandable during a crisis, contributed significantly to the overwhelming workload described by participants. As one nurse noted, managing students with complex needs while handling expanded COVID-19 duties created an unsustainable burden. Other studies echo these findings, with school nurses highlighting that their workload was not only increased but fundamentally changed, with new roles and expectations (e.g., pandemic-related services for students, school staff, parents, community; food distribution, COVID policy development) combined with already full schedules, often without additional support (36, 40, 41). The solutions lie not in expecting nurses to take on more responsibilities, but in building systems that enable them to focus on their core strengths, including clinical care and health promotion. We recommend schools implement team-based approaches, with school staff supporting health initiatives and school nurses delegating appropriate tasks that do not require a registered nurse. Implementing a team-based collaborative approach can help school nurses better fulfill their essential duties and experience less difficulty completing core responsibilities (42).

Despite significant challenges, school nurses demonstrated remarkable resilience, with 53% citing colleague support as a source of hope. This finding underscores the critical importance of peer networks and suggests that interventions targeting professional isolation could yield significant benefits. Nurses who found meaning in their work, drew strength from helping staff and students, shifted their mindset, or relied on faith and spirituality to find hope, exemplified the value of personal resilience. These findings suggest potential for the development of system level programs, such as peer mentoring and support, mental health services, self-care practices, and resilience building to support and sustain school nurse personal resilience (43–45). Organizational support for resilience can enhance nurses' ability to cope with adversity, maintain job satisfaction, and promote long-term wellbeing.

### 4.1 Limitations

This study is not without limitations. This study was conducted in a single, urban-suburban region of California. School funding, public health policies, nurse-to-student ratios, and community demographics can vary across school districts and states. Therefore, our findings may not be fully representative of school nurses in other parts of the United States. Burnout and moral distress were measured using single items. Single-item measures may not be as nuanced as multi-dimensional instruments. However, both the Mini Z burnout item and Moral Distress Thermometer are validated and reliable tools, that are efficient and can reduce burden for study participants (46).

## 5 Conclusion

This study underscores the essential contributions of school nurses to student health, safety, and academic success, particularly during the unprecedented challenges of the COVID-19 pandemic. School nurses demonstrated remarkable adaptability and resilience, providing a wide range of services from pandemic preparedness to chronic health management, often under conditions of high stress and limited support. However, the findings reveal that persistent job stressors, high caseloads, and professional isolation have led to substantial rates of burnout, which not only jeopardizes the wellbeing of school nurses but may also have downstream effects on the quality and safety of student care.

To address these challenges, it is imperative for school systems and policymakers to implement systemic solutions that go beyond expecting nurses to do more with less. Strategies such as increasing staffing, fostering interdisciplinary collaboration, and developing organizational programs to support nurse resilience are critical for sustaining the school nursing workforce. By investing in the wellbeing and empowerment of school nurses, schools can ensure the continuity of high-quality health services and promote healthier, more resilient school communities for the future.

## Data Availability

The datasets presented in this article are not readily available because the datasets generated and analyzed during the current study contain sensitive information from participants regarding their workplaces, mental health, and supervisory relationships. To protect participant privacy and confidentiality, these data are not publicly available. Requests to access the datasets should be directed to Nakia C. Best, PhD, RN, FAAN, nbest@uci.edu.
